# Family policy and food insecurity: an observational analysis in 142 countries

**DOI:** 10.1016/S2542-5196(21)00151-0

**Published:** 2021-08-11

**Authors:** Aaron Reeves, Rachel Loopstra, Valerie Tarasuk

**Affiliations:** a Department of Social Policy and Intervention, University of Oxford, Oxford, UK; b International Inequalities Institute, London School of Economics and Political Science, London, UK; c Department of Nutritional Sciences, King's College London, London, UK; d Department of Nutritional Sciences, University of Toronto, Toronto, ON, Canada

## Abstract

**Background:**

Levels of child malnutrition and hunger across the world have decreased substantially over the past century, and this has had an important role in reducing mortality and improving health. However, progress has stalled. We examined whether family policies (eg, cash transfers from governments that aim to support households with children) are associated with reduced food insecurity.

**Methods:**

In this observational analysis, we used a dataset of individual-level data that captured experience-based measures of food insecurity and sociodemographic characteristics collected by the Gallup World Poll in 142 countries for 2014–17. We then combined this dataset with indicators of the type and generosity of family policies in these countries, taken from the University of California, Los Angeles’ World Policy Analysis Center. We used multilevel regression models to examine the association between the presence of family policies for households with children and the probability of reporting moderate or severe food insecurity or severe food insecurity (moderate or severe food insecurity was defined as a “yes” response to at least four of eight questions on the Gallup Food Insecurity Experience Scale, and severe food insecurity was defined as a “yes” response to at least seven questions). We controlled for multiple covariates, including individual-level measures of social position and country-level measures, such as gross domestic product. We further examined whether this association varied by household income level.

**Findings:**

Using data from 503 713 households, we found that, on average, moderate or severe food insecurity is 4·09 percentage points (95% CI 3·50–4·68) higher in households with at least one child younger than 15 years than in households with no children and severe food insecurity is 2·20 percentage points (1·76–2·63) higher. However, the additional risk of food insecurity among households with children is lower in countries that provide financial support (either means-tested or universal) for families than for countries with little or no financial assistance. These policies not only reduce food insecurity on average, but they also reduce inequalities in food insecurity by benefiting the poorest households most.

**Interpretation:**

In some countries, family policies have been cut back in the past decade and such retrenchment might expose low-income households to increased risk of food insecurity. By increasing investment in family policies, progress towards Sustainable Development Goal 2, zero hunger, might be accelerated and, in turn, improve health for all.

**Funding:**

Wellcome Trust.

## Introduction

Over the past century, a remarkable decrease has been seen in hunger across the world;^1^ however, progress seems to have stalled. Despite a rapid decrease in the proportion of the world's population who were undernourished (ie, whose caloric intake is insufficient to meet their minimum energy requirements) between 2001 and 2011 (from 13·3% to 9·1%), this proportion has changed very little in the years since (8·9% in 2018—ie, almost no change since 2011).^2^ Equally troubling, the proportion of people who are severely food insecure globally (ie, going without food because they are unable to afford it) has increased slightly since 2014.^3^

The Sustainable Development Goals explicitly call on all countries to end hunger, achieve food security, and improve nutrition by 2030. Household food insecurity (ie, insecure and insufficient access to food) affects all countries, albeit to different degrees. For example, the Food and Agriculture Organization (FAO) of the UN's Global Food Insecurity Experience Scale indicates that the proportion of adults with food insecurity in 2018 ranged from 2·7% in Switzerland to 88·5% in Liberia.^3^ However, these estimates mask inequalities in access to food within countries, with food insecurity being concentrated among the most economically deprived. In light of the challenges of further reducing food insecurity among the poorest groups,^4^ some non-government organisations have called for more investment in social protection policies that explicitly address the distributional aspects of food insecurity.^3^

Social protection policies are diverse and can potentially address food insecurity in different ways and for different groups. Cash transfers, social insurance schemes, and labour market interventions might all improve the livelihoods of those in need by reducing income instability and thereby protecting households from financial shocks.^5^ Directly or indirectly, these social protection policies might reduce food insecurity by increasing the amount of money that households have to spend on food.^6^ However, not all of these policies will necessarily improve food security to the same degree and so some have suggested that these programmes must target the households that are most vulnerable to most effectively impact food insecurity.^6^

**Research in context**

**Evidence before this study**

To identify studies investigating the association between family policy and food insecurity, we searched Scopus, Google Scholar, and PubMed on Feb 24, 2021, with no date restrictions, for articles in English using the terms “social protection”, “family policy”, and “cash transfer*” alongside terms referring to food security, including “food insecurity”, “malnutrition”, “undernourishment”, and “underweight” in the abstract or title. We also examined the bibliographies of existing reviews of family policy, nutrition, and health. We identified several country-specific studies that suggest that social protection policies sometimes, but not always, reduce food insecurity. We also identified a small number of cross-national studies in high-income countries that did not specifically capture food insecurity outcomes in response to family policies (most of which were conditional cash transfers of some kind) or uncover these associations across many countries. Furthermore, the socioeconomic consequences of family policy can have varying effects on food budgets and access across household income groups. We did not identify any studies that systematically analysed associations between family policy and individual-level food insecurity outcomes globally among households with children, nor did we find studies examining the distributional consequences of these reforms.

**Added value of this study**

To our knowledge, this is the first systematic analysis of the association between family policies and households’ probabilities of reporting food insecurity across household types and income groups. We used a global dataset of household-level food insecurity indicators, measured through the Food Insecurity Experience Scale developed by the Food and Agriculture Organization of the UN and collected by the Gallup World Poll. These data created a unique opportunity to analyse household-level food insecurity by providing the first survey protocol to measure people's direct experiences of food insecurity at the individual level on a global scale. We combined data from 576 429 household respondents across 142 countries with country-level data on the types of family policy that had been implemented from the University of California, Los Angeles’ World Policy Analysis Center. We used cross-national regression models and a series of additional tests to assess whether our results are explained by other processes. We found that households with children are more likely to experience food insecurity across countries but also that the risk of food insecurity is generally lower in countries with family policies than in countries without family policies.

**Implications of all the available evidence**

Our findings are cause for both optimism and concern among policy makers, donors, international institutions, and medical staff worried about food insecurity, and have particular relevance for those developing family and food insecurity policies. Our results highlight the need to consider how family policies might contribute to reducing hunger and point towards a critical and urgent need for research that assesses the effects of family policy changes on food insecurity among different socioeconomic groups.

One group especially at risk of food insecurity is households with children. This association is driven by the increased rates of poverty among these households and has been observed in many country-level studies of food insecurity^7^, ^8^ and in cross-national research.^4^ Increased rates of poverty among households with children globally^9^ are partly due to the decreased earning trajectories of parents who take time away from work to look after children,^10^ but are also rooted in the increased demands children place on financial resources compared with households without children because of the costs of schooling and child care, leaving less money available for food.

For these reasons, many social protection programmes are specifically targeted at households with children, even if the particular features of the programmes are quite diverse. Some countries provide cash transfers to households with children. In low-income country settings, cash transfers result in increased spending on food, increased food consumption, and reductions in hunger.^6^ Additionally, in high-income countries cash transfers such as child benefits have been linked with reductions in food insecurity,^11^ particularly among low-income single parent families.^12^, ^13^ More generally, a cross-country analysis examined country-level social protection expenditure and food insecurity in 36 Organisation for Economic Co-operation and Development countries, and found a negative association between spending and child food insecurity.^14^

We aimed to determine whether family policies positively affected household food insecurity across the world. We focused on family policies not only because families with children have an increased risk of food insecurity but also because family policies are a widely adopted means of addressing the increased costs associated with having children in the household. If family policies reduce food insecurity, they might be an important tool in making progress towards the goal of ending hunger globally. Building on previous research, we explored whether food insecurity is lower in countries that have implemented family policies and whether these policies primarily benefit families at the bottom of the income distribution.

## Methods

### Study design and data sources

In this observational analysis, we used individual-level data that captured experience-based measures of food insecurity and sociodemographic characteristics from the Gallup World Poll, a survey of stratified random samples in 142 countries (a full list of countries is in the appendix [pp 2–3]), covering the period 2014–17. Interviews are held by telephone in countries where telephone coverage is above 80% and face-to-face everywhere else. Respondents were randomly selected among all members of the household older than 15 years and were sometimes asked to respond on behalf of the whole household, and the presence of children was defined in the Gallup World Poll as members of the household younger than 15 years. More details on the Gallup World Poll methods are in the appendix (p 4).

We merged these data with country-level measures of social policy taken from the University of California, Los Angeles’ World Policy Analysis Center. These measures are largely derived from their poverty database. The family policies included in our analysis are listed in the panel and outlined in detail in the appendix (pp 5–6). We coded countries as 0 if they have not legislated for a given family policy and as 1 if they have legislation in a given area of family policy.

Panel **Income support policies (ie, family policies) for households with children examined in these analyses**

**Income support for families**

Includes cash benefits that are paid directly to households by the government and does not include other types of assistance such as in-kind food assistance or food vouchers (eg, the US Supplemental Nutrition and Assistance Program)*

**Birth or maternity grants**

A birth or maternity grant is a one-time or short-term grant given when a child is born to help with the costs associated with having a child

**Financial support for low-income households with young children**

Cash benefits that are paid directly to households with children aged 4 years by the government

**Financial support for low-income households with school-aged children**

Cash benefits that are paid directly to households with children aged 8 years by the government

**Financial support for low-income households with teenaged children**

Cash benefits that are paid directly to households with children aged 14 years by the government

**Income support for child-care or school costs**

Includes cash benefits to support costs of child care or other school costs (eg, travel and uniforms) that are paid directly to households by the government

The Food Insecurity Experience Scale contained in the Gallup World Poll is a global measure of food insecurity that is intended to provide comparable estimates of multidimensional food insecurity around the world.^4^ It asks a series of eight questions, with answers of “yes” or “no”, that are designed to elicit whether respondents had particular difficulties or uncertainties in consuming sufficient food over the past 12 months, ranging from worry that food supplies would run out and the inability to access healthy and nutritious foods to skipping meals and going without eating. We summed responses across the eight questions (yes = 1, no = 0) and converted the total score into two binary categories of food insecurity as follows:^15^ moderate or severe food insecurity capturing a “yes” response on at least four questions (denoted as food insecure throughout), and severe food insecurity capturing a “yes” response to at least seven questions.

After merging these family policy data with the Gallup World Poll data and other country-level data sources from the World Bank, FAO, and Organisation for Economic Co-operation and Development, we excluded households with missing individual-level and country-level data, reducing the sample size from 576 429 households to 503 713, our analytical sample.

### Statistical models and analysis

To assess the effect of family policy on food insecurity, we estimated separate multilevel, logistic regression models (with random intercepts) for each food insecurity indicator (moderate or severe and severe food insecurity). The absence of policy variation over time precludes exploiting within-country variation. We proceed in three steps.

First, we tested whether the probability of food insecurity for households with children younger than 15 years is higher than for a household with no children younger than 15 years (0 = no child younger than 15 years; 1 = at least one child younger than 15 years in the household).

Second, we estimated whether food insecurity is, on average, lower in countries that have implemented each of the categorised types of family policy than those that have not, as measured by the World Policy Analysis Center. We are interested in the population-level effect of the policies because we know from other work that cash transfers to one household can spillover to other households,^16^ reducing poverty in households that are not directly targeted by the reform.

Third, we tested whether or not the association between family policy and food insecurity is indeed concentrated in households with children. We estimated a cross-level interaction term between whether a child is present in the household and the type of family policy in place. In all our models we estimated marginal effects to provide more accurate estimates of the association between family policy and food insecurity.^17^ We also tested whether these policies are most beneficial for households at the bottom of the income distribution by comparing whether the increased risk of food insecurity due to having a child is reduced more for those in the bottom 40% of the income distribution than for those in the upper 60% of the income distribution. To do this, we added a three-way interaction term into our multilevel logistic regression models between the family policy, whether a household has a child present, and whether household income is in the bottom 40% of the national income distribution. More details are in the appendix (p 8).

For each of these models, we estimated the predicted probability (which can be converted into the population proportion) of being food insecure and then calculated the marginal effect of the policies (including the 95% CIs of these estimates)—ie, the average difference in the predicted probability of being food insecure between countries that have different types of family policies. We calculated p values using our multilevel, logistic regression models and considered p values below 0·05 to be significant.

We adjusted our models for possible confounders, including individual-level variables that might be associated with the presence of children and food insecurity. These confounders were respondent's age (including a squared term), gender, marital status, employment status, and location (rural or urban). Previous work^4^ also suggests measures of social capital might predict food insecurity and so we included two measures of social capital:^4^ whether you have friends or family you can count on and satisfaction with friendships. We also considered country-level confounders that might be associated with countries having family policies in place. We controlled for gross domestic product (GDP) per capita (adjusted for inflation and purchasing power), because richer countries will generally have less food insecurity than poorer countries and will also be more likely to have family policies.^18^, ^19^

In our main models we did not adjust for income because our measure of household income cannot disaggregate between different income sources; however, we did a sensitivity analysis in which we controlled for household income (logged, per household member, and adjusted for purchasing power; appendix p 17). We also did several other sensitivity analyses, including re-estimating our results on a matched sample of countries (pairs were matched by level of economic development [according to the World Bank], population size, degree of democracy [measured using Polity IV], and continent) and accounting for implementation of other policies that might affect food security, such as total government spending on families, presence of free secondary schooling, and the scale of in school feeding programmes (more details of methods in these sensitivity analyses are in the appendix [pp 9–13]). We also did a sensitivity analysis accounting for battle deaths in conflict zones, and we also explored whether food insecurity is lower in countries where at least one income support policy has been implemented and whether implementing additional family policies further reduces food insecurity (appendix pp 14–15). We also adjusted our models for an indicator of the year in which the survey was done (appendix p 16). SEs are clustered at the country level.

All analyses were done using Stata version 15.1.

### Role of the funding source

The funder of the study had no role in study design, data collection, data analysis, data interpretation, or writing of the report.

## Results

Using data from 503 713 respondents from 142 countries for the period 2014–17, we found that households with at least one child younger than 15 years are 4·09 percentage points (95% CI 3·50–4·68) more likely to have moderate or severe food insecurity and 2·20 percentage points (1·76–2·63) more likely to have severe food insecurity than households without children, supporting previous work.^4^

These associations are not trivial. For example, if the prevalence of moderate or severe food insecurity among households with at least one child younger than 15 years was the same as the prevalence of food insecurity among households without a child younger than 15 years, there would be approximately 100 million fewer adults with food insecurity globally (appendix p 7).

Next, we found that, across countries, both forms of food insecurity are lower in countries that have implemented family policies aimed at supporting households with children, adjusted for household type (table). When we looked at specific policies, the results point in a similar direction, albeit with important caveats. The association between each of these policies and food insecurity is consistently negative and even the smallest coefficient suggests food insecurity decreases by over 2 percentage points in the population as a whole where policies are implemented. The policies that provide income support for child care or school costs have 95% CIs that cross zero, which might be due to the small number of countries in low-income countries that have implemented these policies. The association is also weaker for transfers aimed at households with teenage children, at least for moderate or severe food insecurity, than for the other policies.

**Table:** Difference in risk of food insecurity between countries with and without income support for households with children, 2014–17

	**Moderate or severe food insecurity**	**Severe food insecurity**
Income support for families	−7·08 (−2·67 to −11·49; p=0·0016)	−5·96 (−2·67 to −9·24; p=0·0004)
Birth or maternity grants	−6·65 (−2·25 to −11·05; p=0·0031)	−5·20 (−2·05 to −8·35; p=0·0012)
Financial support to low-income households with young children	−6·89 (−2·13 to −11·64; p=0·0045)	−6·09 (−2·53 to −9·64; p=0·0008)
Financial support to low-income households with school-aged children	−6·71 (−1·96 to −11·46; p=0·0057)	−6·08 (−2·55 to −9·62; p=0·0008)
Financial support to low-income households with teenaged children	−3·73 (−8·45 to 0·99; p=0·12)	−3·55 (−0·25 to −6·86; p=0·035)
Income support for child-care or school costs	−3·36 (−9·20 to 2·48; p=0·26)	−2·68 (−7·06 to 1·71; p=0·23)

Data are percentage point changes with 95% CIs in parentheses. Each coefficient comes from a separate regression model. All models were adjusted for the confounders of age, age-squared, gender, marital status, employment status, rural-urban, whether you have friends or family you can count on, satisfaction with friendships, and gross domestic product per capita adjusted for inflation and purchasing power. SEs are clustered at the country level.

The analyses presented so far show population-level associations, but we also tested whether these family policies reduce the risk of food insecurity among their intended beneficiaries—ie, the households with children. We tested this question by adding an interaction term into our multilevel regression models between our different measures of family policy and whether households have a child younger than 15 years. We then calculated the marginal effect of the presence of a child in the household on the risk of food insecurity.

**Figure 1:**
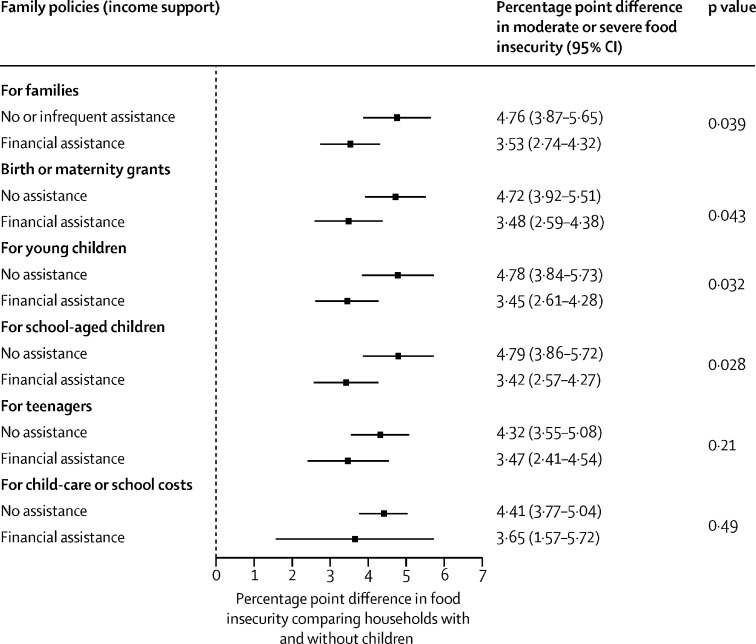
Difference in moderate or severe food insecurity in households with children and without children in countries with and without financial assistance for households with children

**Figure 2:**
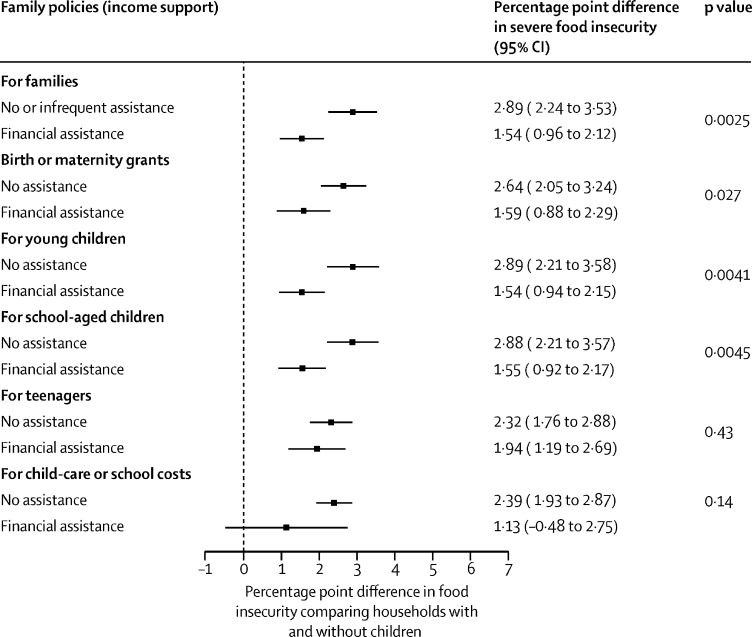
Difference in severe food insecurity between households with and without children in countries with and without financial assistance for households with children

We calculated the change in moderate or severe (figure 1) and severe (figure 2) food insecurity when households had at least one child younger than 15 years in countries with and without specific forms of financial support for households with children. Households with children had a higher risk of food insecurity than those without children, but for almost all family policies this risk was reduced in countries where family policies had been implemented. For severe food insecurity, we found that the relative differences between households with and without the policies in place were larger and the p values were smaller than for moderate and severe food insecurity, suggesting that these policies might be associated with even greater reductions in severe food insecurity than moderate or severe food insecurity (figure 2). However, the association between children and food insecurity was not entirely broken with any of the policies we analysed, although the risk was substantially reduced with the policies in place.

Next, we added a three-way interaction term into our multilevel logistic regression models between the family policy, whether a household has a child present, and whether household income is in the bottom 40% of the national income distribution (appendix p 8). Again, we calculated the marginal effects.^17^ We found that the impact of most of these family policies on whether having children increases food insecurity is greatest for those in the bottom 40% of the income distribution. This observation is true for food insecurity and severe food insecurity. Indeed, in some instances, these policies entirely remove the association between having a child and food insecurity for the poorest households (appendix p 8).

We did a series of robustness checks to explore the sensitivity of our analyses to different specifications. We re-estimated our models after we selected similar countries using a matching procedure. This matching reduced the analytical sample to 91 countries, but we found similar results when using just our matched sample (appendix pp 2–3, 9). Next, using the full sample of countries, we explored whether implementation of family policies might correlate with implementation of other policies that might also affect food insecurity. After constructing a measure of policy context using a principal components analysis of five related policy areas, we added this measure to our regression models as a covariate and found almost exactly the same results as the main analyses (appendix p 10). We also explored whether our results were affected when we accounted for total government spending on families. Importantly, this measure is only available for high-income countries and so provides a strong test of our hypothesis because it focuses on countries that are already similar to each other economically. Although greater government spending on families is associated with lower levels of food insecurity, our measures of family policy remained negatively correlated with food insecurity (appendix p 11). We also found that the availability of free secondary schooling and the scale of school feeding programmes within each country did not affect our findings (appendix pp 12–13). High levels of food insecurity might be associated with conflict zones and so we did a further sensitivity analysis in which we adjusted for battle deaths; however, we found no difference with our main analyses (appendix p 14). Furthermore, we found that food insecurity is lower among countries that have implemented at least one policy compared with countries that have implemented none, but it is lower still in countries that have implemented more of these policies (appendix p 15). We also explored whether controlling for time dummies affected our results, and found that they did not (appendix p 16).

In these analyses, we have not accounted for household income because the data we used only contain a measure of total income, which could bias our results if respondents included income from cash transfers in their answer. However, ignoring household income completely raises the possibility that our analyses were not accounting for the main determinant of food insecurity. Finally, we estimated our main models again controlling for household income, and found that the results remained consistent with our main analyses (appendix p 17).

## Discussion

Ending hunger and achieving food security means ensuring all people, at all times, have access to a sufficient quantity and quality of food to ensure good health. Progress towards this goal has stalled, such that many have inadequate access to food and all of the health consequences that accompany this situation. This is especially worrying because, as confirmed by our analysis, food insecurity is more common among households with children.^4^

We examined whether social policies specifically intended to reduce poverty among households with children are associated with reduced food insecurity. Three findings emerged from our analysis. First, countries that have implemented family policies have, on average, lower food insecurity than those that have not. This difference can be illustrated by comparing two small west African countries: Sierra Leone and Togo. According to the World Bank, in Sierra Leone approximately 84% of the population is food insecure, while in Togo approximately 68% of the population is food insecure, despite similar levels of economic development (purchasing power parity $1794 *vs* $1667, in international $) and population (8 million *vs* 8·3 million). Sierra Leone has no known family cash benefits whereas Togo provides cash benefits to households with children without a means test. Although many important differences exist between these two countries, including potential imperfections in how Togo's scheme is delivered, our results suggest that the level of food insecurity in Sierra Leone could be reduced if the government instituted the same kind of family policies as in Togo.

Beyond these population-level associations, we also found that family policies are associated with reduced food insecurity among specific groups, including families with children and those at the bottom of the income distribution. Hence, social protection potentially addresses inequalities in food insecurity across households, especially if the administration of these policies is clear and well organised.^20^ However, notably, our results do not imply that all programmes are equally effective, and countries with weaker state capacity might be less able to ensure these funds reach the families that need them the most.^21^

Despite these uncertainties in the analysis, our cross-national results substantially advance our understanding of the contribution of social protection policies to lowering food insecurity around the world. Although earlier work has found that the introduction of child-specific income transfers has been associated with reductions in food insecurity in single case-study countries,^6^, ^12^, ^22^ our results suggest that these findings are generalisable to many countries. These findings are also important because they suggest that income-transfer policies specifically benefit families with children at the bottom of the income distribution and they reduce the most severe forms of food insecurity. Our results contribute to global evidence on the role of social safety nets in reducing extreme poverty and improving the ability of households to meet basic needs.^23^ Additionally, our findings are important because they highlight that these households have the highest risks of the most severe health consequences of food insecurity, including wasting and stunting, nutrient deficiencies, and poor mental health. Our research reinforces evidence suggesting that increased investments in social protection lead to improved health outcomes.^24^, ^25^ Indeed, one of the key mechanisms behind these associations might be that social protection reduces food insecurity.

Our findings might also contribute to wider debates about the implications of welfare retrenchment for health and poverty reduction. The recent history of family policies has not been one of straightforward progress, with some countries strengthening their commitment to households with children, and others weakening the level of support they offer to families in economically precarious situations.^26^, ^27^ Both strengthening and weakening family policies—irrespective of the level of GDP—are likely to have implications for households’ access to food. For example, food insecurity is reduced when cash transfers to families are more generous.^23^, ^28^ Reforms in both Canada and the USA that increased the level of financial support offered to low-income households through the social security system have directly led to reduced food insecurity.^11^, ^12^ But the contrary also seems to be true, with reduced generosity increasing food security. Food insecurity might increase when cash transfers are reduced,^12^, ^29^ and so high-income countries cannot simply assume that their increased economic development is sufficient to end hunger for those in need.^18^, ^19^ Some evidence suggests that cash transfers to households with children might be especially effective in low-income and middle-income countries.^6^ In this respect, the history of food insecurity in high-income countries should be used as an example of the possible consequences of cutting family policies.

Our analysis has some important limitations. Although we report data measuring food insecurity in a large number of countries, these data only cover a short period of time and so we have not examined policy change. This limitation is important because we were not able to analyse the impact of implementing these policies on food insecurity. Additionally, the quality of data almost certainly varies across countries, despite the standardised procedures used by the Word Gallup Poll, and this might bias our results. Additionally, we were not able to address differences in the generosity of benefits nor how these policies were implemented. Statutory commitments might be in place but whether the cash actually reaches the people who need it is not captured by our data. Although having these policies institutionalised seems to reduce food insecurity for the average household with children, more work is needed to assess whether implementation fidelity affects the extent to which they reduce food insecurity for all families. We also recognise that although the policies we analysed are cash transfers of some kind, a wide range of policy instruments that could be included under each policy domain exist. We are also cautious about drawing strong conclusions regarding the policies aimed at households with teenaged children and those covering child care or other education-related costs. The null findings we report here might be because low-income countries are less likely to provide support for child-related costs. Our main results do not account for household income because it is impossible to separate out income from the policies we are studying from other sources of income. Although we explore our results after adjusting for total income, more work is needed to understand how these policies interact with different sources of income. Finally, as highlighted in the Methods section, our estimates might also be biased towards the null because they do not account for regional variation in child policies (ie, across subnational forms of government); more work is needed in these areas.

Family policies are not a panacea for solving food insecurity, even among households with children. Economic growth has been responsible for great improvements in living conditions for many people around the world, and it makes investments in the economically vulnerable more sustainable.^30^ But, in light of the high levels of food insecurity among households with children around the world, even in high-income countries,^4^, ^8^, ^11^ policy makers must look for other mechanisms to address the challenge of eliminating food insecurity among the 2 billion people and 600 million children who do not have regular access to safe, nutritious, and sufficient food.^31^ Cash benefits for families might have a crucial role in these efforts to end food insecurity.

## Data sharing

The data used in this study will not be made available to other researchers. The data are proprietary and owned by the survey company, Gallup. We obtained access to it through a special license provided by the FAO. However, the statistical code used to produce the results is available online.

## Declaration of interests

We declare no competing interests. The Food Insecurity Experience Scale was collected as part of the Gallup World Poll. The FAO commissioned Gallup to include this scale on their survey and purchased a number of licenses to share with researchers. Our research team applied for access to the data and were awarded one of these licenses.

## Footnotes

* All policies examined do not include other types of assistance such as in-kind food assistance or food vouchers; more details and illustrative polices are in the appendix (pp 5–6).
