# Supplementary material for: Family policy and food insecurity: an observational analysis in 142 countries
Source: Lancet Planet Health. 2021 Aug 11;5(8):e506–13. doi: 10.1016/S2542-5196(21)00151-0 (PMC8374132; doi:10.1016/S2542-5196(21)00151-0)
Supplement: Supplementary appendix [file mmc1.pdf]

THE LANCET
Planetary Health

Supplementary appendix

This appendix formed part of the original submission and has been peer reviewed. We post it as supplied by the authors.

Supplement to: Reeves A, Loopstra R, Tarasuk V. Family policy and food insecurity: an observational analysis in 142 countries. *Lancet Planet Health* 2021; **5:** e506–13.

Web Appendix

Web Appendix 1: Countries included in the main analysis

Web Appendix 2: Methodological details of the Gallup World Poll

Web Appendix 3: Background information on the data from the World Policy Analysis Center and the family policy measures included in the analysis

Web Appendix 4: Counterfactual calculation of difference in food insecurity

Web Appendix 5: Estimating the risk of food insecurity in countries with generous family policies for households with children and who are in poverty

Web Appendix 6: Estimating the risk of food insecurity in countries with generous family policies in a matched set of countries

Web Appendix 7: Estimating the risk of food insecurity after accounting for other social protection policies implemented in the same country

Web Appendix 8: Income support policies for households with children reduce the risk of food insecurity in 34 OECD countries and adjusting for government spending on families as a proportion of GDP.

Web Appendix 9: Income support policies for households with children reduce the risk of food insecurity adjusting for the availability of free secondary schooling.

Web Appendix 10: Income support policies for households with children reduce the risk of food insecurity adjusting for the availability and scale of school feeding programs.

Web Appendix 11: Income support policies for households with children reduce the risk of food insecurity adjusting for the number of battle deaths over the last 40 years.

Web Appendix 12: Food insecurity is lower in countries that have implemented (a) any of these policies and (b) more of these policies

Web Appendix 13: Income support policies for households with children reduce the risk of food insecurity, adjusting for time dummies.

Web Appendix 14: Income support policies for households with children reduce the risk of food insecurity, adjusting for household income.

Web Appendix 1: Countries included in the main analysis

Country	N	Including in matching analysis	Country	N	Including in matching analysis
Afghanistan	3654		Kyrgyzstan	3764	
Albania	3836	X	Laos	929	X
Algeria	2804	X	Latvia	3460	
Angola	870	X	Lebanon	3915	X
Argentina	3633	X	Lesotho	1885	X
Armenia	3668	X	Liberia	3780	
Australia	3886		Lithuania	3008	
Austria	3799		Luxembourg	3922	
Azerbaijan	3337	X	Macedonia	3815	X
Bahrain	3969		Madagascar	3957	X
Bangladesh	3910		Malawi	3985	X
Belarus	3566	X	Malaysia	1929	X
Belgium	3978		Mali	3913	X
Belize	478		Malta	3872	X
Benin	3698	X	Mauritania	3742	X
Bhutan	1997		Mauritius	2864	X
Bolivia	3699	X	Mexico	3839	X
Bosnia Herzegovina	3630		Moldova	2649	X
Botswana	3844	X	Montenegro	3747	X
Brazil	3952	X	Morocco	3858	X
Bulgaria	3677	X	Myanmar	4502	
Burkina Faso	3853	X	Namibia	1940	X
Burundi	995	X	Nepal	3918	
Cambodia	4553		Netherlands	3936	
Cameroon	2868	X	New Zealand	3889	
Canada	3087	X	Nicaragua	3670	X
Chad	3868	X	Niger	3822	X
Chile	3818	X	Nigeria	3895	
China	3721	X	Norway	3900	
Colombia	3865	X	Pakistan	4490	X
Congo Brazzaville	3674	X	Panama	3571	X
Congo Kinshasa	3849	X	Paraguay	3748	X
Costa Rica	3485	X	Peru	3834	X
Croatia	3561		Philippines	3959	X
Cyprus	3832		Poland	3580	
Czech Republic	3621		Portugal	3835	
Denmark	3877		Romania	3615	X
Dominican Republic	3658	X	Russia	6789	
Ecuador	3880	X	Rwanda	3960	X
Egypt	3928		Saudi Arabia	3891	X
El Salvador	3517	X	Senegal	3922	X
Estonia	3449		Serbia	3582	X
Ethiopia	3935		Sierra Leone	3851	X
Finland	3904		Singapore	2833	
France	3891		Slovakia	3694	
Gabon	3827	X	Slovenia	3913	
Gambia	934		South Africa	3923	X
Georgia	3682	X	South Korea	3602	

Germany	3760		Spain	1961	
Ghana	3854	X	Sri Lanka	3044	X
Greece	3865		Sudan	784	X
Guatemala	3541	X	Sweden	3860	
Guinea	3853	X	Switzerland	3403	
Haiti	1704		Tanzania	3890	X
Honduras	3489	X	Thailand	2924	X
Hungary	3507	X	Togo	3777	X
Iceland	1526	X	Trinidad and Tobago	455	X
India	11426	X	Tunisia	3848	X
Indonesia	3906	X	Turkey	2841	X
Iran	3847	X	Uganda	3863	X
Iraq	3867	X	Ukraine	3264	X
Ireland	3930		United Arab Emirates	6173	X
Israel	3576	X	United Kingdom	3912	
Italy	3925		United States	2923	
Ivory Coast	3842		Uruguay	3475	X
Jamaica	899	X	Uzbekistan	3857	X
Japan	3636		Venezuela	3861	X
Jordan	3963	X	Vietnam	2851	X
Kazakhstan	3502	X	Yemen	3903	X
Kenya	3984	X	Zambia	3883	X
Kuwait	3734		Zimbabwe	3911	X

Web Appendix 2: Methodological details of the Gallup World Poll

The Gallup World Poll (GWP) is a high quality survey that is being used by the United Nations to track progress toward the Sustainable Development Goals. Whilst there is not a category of ‘official statistics’ at the global level, the GWP is as close as we get. It consists of a stratified random sample. The data has been used by a wide number of studies. We are using data from 142 of the surveyed countries (see Appendix 1 for full list) and the data covers 2014 to 2017.

Gallup typically aims to survey approximately 1000 people in each country although this does vary (see Appendix 1). Face-to-face interview are approximately 1 hour while telephone interviews last around 30 minutes. They survey is usually conducted once per year and fieldwork is typically conducted in less than one month.

Almost all samples are probability based and are nationally representative of the adult population. The sampling frame represents the entire civilian, non-institutionalized, aged 15 and older population. There are exceptions, of course, and these include areas where the safety of interviewing staff is threatened (e.g., conflict zones), scarcely populated islands in some countries, and areas that interviewers can reach only by foot, animal, or small boat.

Interviews are conducted by telephone in countries where telephone coverage is >80% and face-to-face everywhere else. Respondents are randomly selected among all members of the household over the age of 15, and the presence of children is defined in the GWP as members of the household under the age of 15.

In countries where there is face-to-face surveying, the first stage of sampling is the identification of 100-135 sampling units. These units are stratified by population size and or geography. Sample selection is based on probabilities proportional to population size, otherwise simple random sampling is used. Random route procedures are used to select sampled households. Finally, respondents are then randomly selected within the selected households.

In countries where there is telephone interviewing, random-digit-dialling or a nationally representative list of phone numbers is used. Where mobile phone use is high, a dual sampling frame is used. Each selected individual receives three attempts to reach them, spread over different days and times of the day.

The survey is translated in the major conversational languages of each country but starts with either the English, French, or Spanish version depending on the region. Each translation is validated by two people.

Gallup World Poll response rates vary by mode of survey and region with an average response rate of 80% in Sub Saharan Africa to 50% in former Soviet Union countries. These response rates are high compared to other similar surveys.

Web Appendix 3: Background information on the data from the World Policy Analysis Center and the family policy measures included in the analysis

The World Policy Analysis Center database is constructed from the constitutional and legal provisions for families with children. The coding is based on official documents and if these are unavailable the data are derived from country reports to UN agencies. The basic sources for the policy data come from Legislative texts accessed via the ILO's NATLEX database and official government gazettes; Social Security Programs Throughout the World (SSPTW) reports; the ILO's TRAVAIL Database of Conditions of Work and Employment Laws; the European Union's Mutual Information System on Social Protection; and the Mutual Information System on Social Protection of the Council of Europe.

There are three notable limitations to these data. First, only family benefits provided on a statutory basis are captured in this data. This may mean that there are non-statutory benefits offered to households which are not formally included in this data set, such as funds made available by local government on an *ad hoc* basis. Second, country reports to UN agencies may introduce bias because countries may declare that they are doing more than they in fact are.[32] This bias is likely to move in a single direction, that is, we think it unlikely countries say they are doing less than they really are, and so our results are likely to be conservative estimates because there will be some countries who should be in the untreated category that are in measured as treated. The third limitation is that there may be regional variation in family policies within countries that are ignored by these country-level measures. These measures constitute the social protection floor for families at the country-level.

Some of these benefits (*Income support for families* and *Income support for childcare or school costs)* are provided after a means test, that is after the government or some other agency have assessed the amount of financial resources available to the family. Typically, if households fall below a particular income level then they are deemed eligible to receive the support. Universal or unconditional benefits are offered to everyone irrespective of their level of income.

Below we also provide some examples of specific policies that might be categorised under each heading. This elaborates on the information included in Box 1.

Income support for families: This includes cash benefits which are paid directly to households by the government. This includes measures such as 'Allocation familiale' in Algeria or Benin's 'Allocation familiale' (which is paid to all children under the age of 22). But it does *not* include other types of assistance such as in-kind food assistance or food vouchers (e.g. the U.S. Supplemental Nutrition and Assistance Program). We code countries that do not offer such support or only do so in some circumstances, such as to orphans, as 0 and countries which offer this support conditional on a means test or to everyone irrespective of their economic circumstances are coded as 1.

Birth or maternity grants: A birth or maternity grant is a one-time or short-term grant given when a child is born to help with the costs associated with having a child. Countries where no grant is available are coded as 0 while countries with any birth or maternity grant are coded as 1. This includes measures such as the 'Newborn upfront payment' in Australia or the Bahamas' 'Maternity Grant'.

Financial support to low-income households with young children (~4 and under): This includes cash benefits which are paid directly to households by the government and does not include other types of assistance such as in-kind food assistance or food vouchers. Countries where no support is available are coded as 0 while countries with any level of support are coded as 1.

Financial support to low-income households with school-aged children (~5-12yo): This includes cash benefits which are paid directly to households by the government and does not include other types of assistance such as in-kind food assistance or food vouchers. Countries where no support is available are coded as 0 while countries with any level of support are coded as 1. This includes Brazil's 'Family Allowance' (which is only paid to children under 14) and Cameroon's 'Family Allowance (which is only paid unconditionally to children under the age of 6).

Financial support to low-income households with teenaged children (~13-19yo): This includes cash benefits which are paid directly to households by the government and does not include other types of assistance such as in-kind food assistance or food vouchers. Countries where no support is available are coded as 0 while countries with any level of support are coded as 1. This includes the 'Child support benefit' in Greece (which covers children up to 18) and Belgium's 'Family Allowance' which covers all children unconditionally until the age of 18.

Income support for childcare or school costs: This includes cash benefits which are paid directly to households by the government. We code countries that do not offer such support as 0, and we code countries as 1 if they offer some support for childcare or other school costs (such as travel or the cost of uniforms). This includes the 'Childcare subsidy' in Australia or 'Child care benefit' in Azerbaijan.

Web Appendix 4: Counterfactual calculation of difference in food insecurity

We calculate this figure in the following way. Our sample covers 99% of the world's population and about 48.68% of households have no children under 15. We then take the marginal probability of being moderately or severely food insecure for both sets of households (0.2474 for households without children and 0.2870 for households with children) and calculate the difference in the proportion of people who are food insecure if households with children had the same probability of being food insecure as households without children (0.2870 to 0.2474). Our simple estimate suggests food insecurity would be ~2% smaller, which is approximately 113 million households. This is a conservative estimate not only because we round the number down but also because it assumes there is no spill-over to other members of the household, such as children.

Web Appendix 5: Estimating the risk of food insecurity in countries with generous family policies for households with children and who are in poverty

Here we estimate the marginal association between having a child for 4 groups of people:

1. Households in the bottom 40% of the income distribution in countries without the policy in question (see table below).
2. Households in the bottom 40% of the income distribution in countries with the policy in question (see table below).
3. Households in the upper 60% of the income distribution in countries without the policy in question (see table below).
4. Households in the upper 60% of the income distribution in countries with the policy in question (see table below).

We then calculate the difference in the marginal effect of having a child for those in similar economic positions but who are in different policy contexts, so marginal effect for group 1 – marginal effect for group 2, and marginal effect for group 3 – marginal effect for group 4.

Finally, we calculate whether the difference in these marginal effects: (1 – 2) – (3 – 4), and calculate the p-value for this contrast. Higher values imply that the impact of the policy is higher for poorer households.

Family Policies	Moderate or severe food insecurity (SE)	Severe food insecurity (SE)
	(1)	(2)
Income support for families	2.8%** (0.09)	3.1%** (0.07)
Birth or maternity grants	2.8%** (0.10)	2.8%** (0.08)
Financial support to low-income households with young children	2.2%* (0.11)	2.7%** (0.08)
Financial support to low-income households with school-aged children	2.4%* (0.11)	2.8%** (0.08)
Financial support to low-income households with teenaged children	1.6% (0.13)	2.6%** (0.10)
Income support for child-care or school costs	-0.06% (0.14)	-0.04% (0.12)

Notes: Each coefficient comes from a separate regression model. All models include age, age-squared, gender, marital status, employment status, rural-urban, whether you have friends or family you can count on, satisfaction with friendships, and GDP per capita adjusted for inflation and purchasing-power. Standard errors are clustered at the country-level. * $p < 0.05$, ** $p < 0.01$

Web Appendix 6: Estimating the risk of food insecurity in countries with generous family policies in a matched set of countries

Countries that have and have not implemented generous family policies may be different in other ways that could be correlated with the risk of food insecurity. We therefore implement a matching procedure as a way of trimming outlier countries from our analysis, thereby focussing on the overlapping parts of the distribution of countries with and without generous family policies. We match on the level of economic development (according to the World Bank), population size, the degree of democracy, and their continent using Coarsened Exact Matching [33]. This leaves us with 91 countries.

We estimate both a straightforward matching model and a doubly robust matching model. The difference between the two is that in the doubly robust matching model we also include the covariates in the statistical model that were used to create the matching model. This allows us to capture variation within the matched sample too.

	Matching model		Doubly robust matching	
	Moderate or severe food insecurity	Severe food insecurity	Moderate or severe food insecurity	Severe food insecurity
	(1)	(2)	(3)	(4)
Income support for families	-11.14%**<br>(4.07)	-9.69%**<br>(3.01)	-9.95%**<br>(2.64)	-8.22%**<br>(1.94)
Birth or maternity grants	-10.30%*<br>(4.45)	-8.43%**<br>(3.11)	-4.71%<br>(3.36)	-3.79%<br>(2.60)
Financial support to low-income households with young children	-12.76%**<br>(4.34)	-11.29%**<br>(3.21)	-10.37%**<br>(3.20)	-8.95%**<br>(2.30)
Financial support to low-income households with school-aged children	-12.03%**<br>(4.37)	-10.91%**<br>(3.19)	-10.88%**<br>(3.31)	-9.59%**<br>(2.34)
Financial support to low-income households with teenaged children	-11.01%*<br>(4.83)	-9.16%**<br>(3.17)	-1.75%<br>(4.08)	-2.31%<br>(2.95)
Income support for child-care or school costs	-15.77%**<br>(4.63)	-9.79%<br>(3.31)	-4.02%<br>(4.68)	-1.99%<br>(3.30)

Notes: Each coefficient comes from a separate regression model. All models include age, age-squared, gender, marital status, employment status, rural-urban, whether you have friends or family you can count on, satisfaction with friendships, GDP per capita adjusted for inflation and purchasing-power, and government spending on families as a proportion of GDP. Standard errors are clustered at the country-level. Doubly robust models includes the covariates into the model itself in addition to selecting the matched countries. * $p < 0.05$, ** $p < 0.01$

Web Appendix 7: Estimating the risk of food insecurity after accounting for other social protection policies implemented in the same country

The differences we observe in food insecurity between countries with strong and weak family policies could be because countries with strong family policies are more likely to implement other social protection policies which also affect food insecurity. We first construct a measure of policy context through taking the principle components of 4 policy areas: minimum wage policy, the generosity of unemployment insurance, the income protections for the elderly, and the income protections for families with disabled children. Over 65% of the variance is explained by the first component, suggesting that these variables are all largely correlated with each other. We then implement an orthogonal rotation of the loading matrix and predicted the scores for the first component. We then add this measure of policy context to our model as a covariate.

	Moderate or severe food insecurity	Severe food insecurity
	(1)	(4)
Income support for families x Households with children	-1.14%<br>(0.6)	-1.32%**<br>(0.5)
Income support for families	-6.32%*<br>(2.68)	-5.60%**<br>(2.05)
Household with at least one child under the age of 15	4.09%**<br>(0.32)	2.17%**<br>(0.23)
Socio-demographic controls	Y	Y
GDP per capita	Y	Y
Measure of Policy Context	Y	Y
Number of people	405,208	405,208
Countries	115	115

Notes: Constant estimated but not reported. Standard errors are clustered at the country-level. All models include age, age-squared, gender, marital status, employment status, rural-urban, whether you have friends or family you can count on, satisfaction with friendships, GDP per capita adjusted for inflation and purchasing-power, and a summary score of the other social protection policies implemented in the same country. * $p < 0.05$, ** $p < 0.01$

Web Appendix 8: Income support policies for households with children reduce the risk of food insecurity in 34 OECD countries and adjusting for government spending on families as a proportion of GDP.

	Moderate or severe food insecurity	Severe food insecurity
	(1)	(2)
Income support for families	-5.15%**<br>(1.04)	-2.45%**<br>(0.57)
Birth or maternity grants	-1.46%<br>(1.03)	-0.97%*<br>(0.47)
Financial support to low-income households with young children	-4.86%**<br>(1.10)	-2.23%**<br>(0.69)
Financial support to low-income households with school-aged children	-4.79%**<br>(1.10)	-2.20%**<br>(0.68)
Financial support to low-income households with teenaged children	-1.87%*<br>(0.84)	-0.84%*<br>(0.42)
Income support for child-care or school costs	0.27%<br>(0.92)	0.028%<br>(0.44)

Notes: Each coefficient comes from a separate regression model. All models include age, age-squared, gender, marital status, employment status, rural-urban, whether you have friends or family you can count on, satisfaction with friendships, GDP per capita adjusted for inflation and purchasing-power, and government spending on families as a proportion of GDP. Standard errors are clustered at the country-level. * $p < 0.05$, ** $p < 0.01$

Web Appendix 9: Income support policies for households with children reduce the risk of food insecurity adjusting for the availability of free secondary schooling.

	Moderate or severe food insecurity	Severe food insecurity
	(1)	(2)
Income support for families	-7.78%**<br>(2.37)	-6.49%**<br>(1.75)
Birth or maternity grants	-6.39%*<br>(2.49)	-5.16%**<br>(1.78)
Financial support to low-income households with young children	-7.37%**<br>(2.60)	-6.41%**<br>(1.92)
Financial support to low-income households with school-aged children	-7.13%**<br>(2.58)	-6.39%**<br>(1.91)
Financial support to low-income households with teenaged children	-3.78%<br>(2.33)	-3.55%*<br>(1.71)
Income support for child-care or school costs	-2.95%<br>(2.88)	-2.45%<br>(2.19)

Notes: Each coefficient comes from a separate regression model. All models include age, age-squared, gender, marital status, employment status, rural-urban, whether you have friends or family you can count on, satisfaction with friendships, GDP per capita adjusted for inflation and purchasing-power, and free secondary schooling. Standard errors are clustered at the country-level. * $p < 0.05$, ** $p < 0.01$

Web Appendix 10: Income support policies for households with children reduce the risk of food insecurity adjusting for the availability and scale of school feeding programs.

Our measure of school feeding programs is taken from Wood Food Program data. Their data contains measures of the number of pre-primary-, primary- and secondary-school children who receive some form of school feeding from the national school feeding programme. This is not necessarily very extensive and may only include biscuit or snack. Beneficiary figures were obtained from several sources including the WFP global school feeding survey. Sometimes these data are estimated when the information could not be obtained in other ways. The data used here is for 2011. We constructed a measure of the proportion of the population receiving these meals. While we acknowledge that these reforms do not benefit everyone in the population, countries with higher population coverage still represent contexts in which these schemes have a larger impact.

	Moderate/severe food insecurity	Severe food insecurity
	(1)	(2)
Income support for families	-7.16%**<br>(-2.65 to -11.67)	-6.04%**<br>(-2.78 to -9.29)
Birth or maternity grants	-6.68%**<br>(-2.46 to -10.90)	-5.02%**<br>(-1.99 to -8.05)
Financial support to low-income households with young children	-6.87%**<br>(-1.88 to -11.85)	-6.16%**<br>(-2.56 to -9.75)
Financial support to low-income households with school-aged children	-6.75%**<br>(-1.80 to -11.69)	-6.20%**<br>(-2.65 to -9.74)
Financial support to low-income households with teenaged children	-1.00%<br>(-5.88 to 3.89)	-1.65%<br>(-5.14 to 1.85)
Income support for child-care or school costs	-3.89%<br>(-8.75 to 0.98)	-2.89%<br>(-6.75 to 0.96)

Notes: Each coefficient comes from a separate regression model. All models include age, age-squared, gender, marital status, employment status, rural-urban, whether you have friends or family you can count on, satisfaction with friendships, GDP per capita adjusted for inflation and purchasing-power, and a measure of the proportion of people covered by school feeding programs. Standard errors are clustered at the country-level. Confidence intervals are reported in parentheses. * $p < 0.05$, ** $p < 0.01$

Web Appendix 11: Income support policies for households with children reduce the risk of food insecurity adjusting for the number of battle deaths over the last 40 years.

We sum the best estimate of the battle-related deaths (taken from the Uppsala Conflict Data Program) for each country since the 1970s and then scale the number of casualties by the current population. We do this because we want to create measure which captures some dimension of the degree of disruption associated with the conflict and our assumption here (albeit imperfect) is that more deaths as a proportion of current population gives us some indication of how widespread and disruptive this conflict has been.

	Moderate/severe food insecurity	Severe food insecurity
	(1)	(2)
Income support for families	-7.28%**<br>(-2.80 to -11.77)	-6.16%**<br>(-2.82 to -9.50)
Birth or maternity grants	-6.76%**<br>(-2.32 to -11.20)	-5.32%**<br>(-2.16 to -8.48)
Financial support to low-income households with young children	-7.05%**<br>(-2.13 to -11.98)	-6.38%**<br>(-2.67 to -10.07)
Financial support to low-income households with school-aged children	-6.86%**<br>(-1.95 to -11.78)	-6.37%**<br>(-2.70 to -10.03)
Financial support to low-income households with teenaged children	-3.73%<br>(-10.10 to 0.85)	-3.61%*<br>(-0.29 to -6.93)
Income support for child-care or school costs	-3.41%<br>(-9.25 to 2.42)	-2.75%<br>(-7.11 to 1.62)

Notes: Each coefficient comes from a separate regression model. All models include age, age-squared, gender, marital status, employment status, rural-urban, whether you have friends or family you can count on, satisfaction with friendships, GDP per capita adjusted for inflation and purchasing-power, and a measure of the proportion of people covered by school feeding programs. Standard errors are clustered at the country-level. Confidence intervals are reported in parentheses. * $p < 0.05$, ** $p < 0.01$

Web Appendix 12: Food insecurity is lower in countries that have implemented (a) any of these policies and (b) more of these policies

A. Whether any of these policies have been implemented

	Moderate or severe food insecurity	Severe food insecurity
	(1)	(2)
Whether a country has implemented any of family policy measures	-8.64%**<br>(2.72)	-7.02%**<br>(2.05)

Notes: Each coefficient comes from a separate regression model. All models include age, age-squared, gender, marital status, employment status, rural-urban, whether you have friends or family you can count on, satisfaction with friendships, household income (per capita, adjusted for purchasing power), and GDP per capita adjusted for inflation and purchasing-power. Standard errors are clustered at the country-level. * $p < 0.05$, ** $p < 0.01$

B. Number of policies that have been implemented

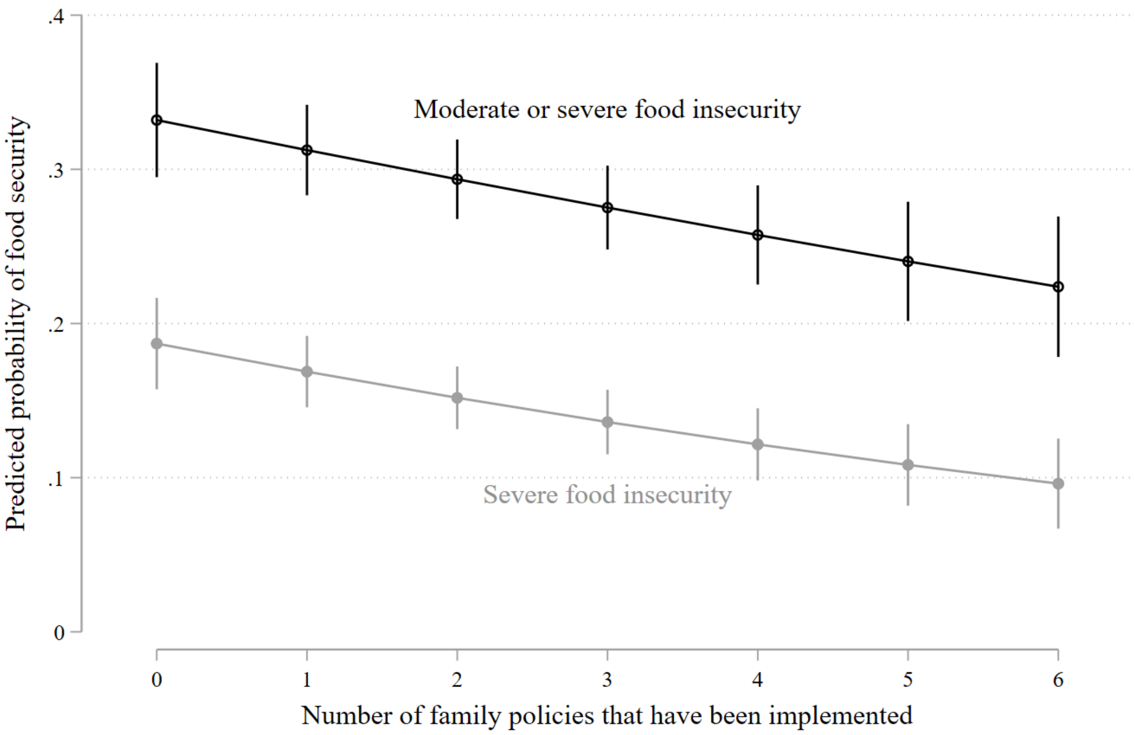

Notes: Estimates come from a multilevel logistic regression model similar the ones estimated in table 2. The primary difference is that instead of estimating a dummy variable indicating the presence of one policy we include a linear variable which measures the number of these policies a country has implemented. The model suggests that countries which have implemented more of these policies have lower food insecurity.

Web Appendix 13: Income support policies for households with children reduce the risk of food insecurity, adjusting for time dummies.

	Moderate/severe food insecurity	Severe food insecurity
	(1)	(2)
Income support for families	-6.89%**<br>(-2.09 to -11.68)	-5.95%**<br>(-2.42 to -9.47)
Birth or maternity grants	-6.88%**<br>(-2.21 to -11.55)	-5.65%**<br>(-2.32 to -8.98)
Financial support to low-income households with young children	-6.71%**<br>(-1.83 to -11.60)	-5.92%**<br>(-2.30 to -9.54)
Financial support to low-income households with school-aged children	-6.53%**<br>(-1.67 to -11.40)	-5.89%**<br>(-2.30 to -9.48)
Financial support to low-income households with teenaged children	-4.06%<br>(-9.10 to 0.99)	-3.85%*<br>(-0.35 to -7.34)
Income support for child-care or school costs	-4.60%<br>(-10.61 to 1.41)	-3.63%<br>(-8.02 to 0.77)

Notes: Each coefficient comes from a separate regression model. All models include age, age-squared, gender, marital status, employment status, rural-urban, whether you have friends or family you can count on, satisfaction with friendships, GDP per capita adjusted for inflation and purchasing-power, and a measure of the proportion of people covered by school feeding programs. All models adjust for time dummies. Standard errors are clustered at the country-level. Confidence intervals are reported in parentheses. * $p < 0.05$, ** $p < 0.01$

Web Appendix 14: Income support policies for households with children reduce the risk of food insecurity, adjusting for household income.

	Moderate or severe food insecurity	Severe food insecurity
	(1)	(2)
Income support for families	-6.40%** (1.96)	-5.36%** (1.42)
Birth or maternity grants	-5.20%** (1.94)	-4.19%** (1.36)
Financial support to low-income households with young children	-6.48%** (2.11)	-5.63%** (1.54)
Financial support to low-income households with school-aged children	-6.35%** (2.11)	-5.66%** (1.53)
Financial support to low-income households with teenaged children	-3.40% (2.02)	-3.18%* (1.39)
Income support for child-care or school costs	-3.00% (2.55)	-2.34% (1.89)

Notes: Each coefficient comes from a separate regression model. All models include age, age-squared, gender, marital status, employment status, rural-urban, whether you have friends or family you can count on, satisfaction with friendships, household income (per capita, adjusted for purchasing power), and GDP per capita adjusted for inflation and purchasing-power. Standard errors are clustered at the country-level. * $p < 0.05$, ** $p < 0.01$
